# Mechanisms underlying reversed TRAIL sensitivity in acquired bortezomib-resistant non-small cell lung cancer cells

**DOI:** 10.20517/cdr.2024.14

**Published:** 2024-04-09

**Authors:** Leonie De Wilt, Bartosz Kamil Sobocki, Gerrit Jansen, Hessan Tabeian, Steven de Jong, Godefridus J. Peters, Frank Kruyt

**Affiliations:** ^1^Department of Medical Oncology, Amsterdam University Medical Centers, Location VUMC, Vrije Universiteit Amsterdam, Amsterdam 1007MB, the Netherlands.; ^2^Department of Biochemistry, Medical University of Gdańsk, Gdańsk 80-210, Poland.; ^3^Department of Rheumatology, Amsterdam University Medical Centers, Vrije Universiteit Amsterdam, Amsterdam 1081 HV, the Netherlands.; ^4^Department of Medical Oncology, University of Groningen, University Medical Center Groningen, Groningen 9713 GZ, the Netherlands.; ^#^Authors contributed equally.

**Keywords:** TRAIL, bortezomib, resistance, sensitization, Bcl-2 family, lipid rafts, cytokines

## Abstract

**Aim:** The therapeutic targeting of the tumor necrosis factor (TNF)-related apoptosis-inducing ligand (TRAIL) death receptors in cancer, including non-small cell lung cancer (NSCLC), is a widely studied approach for tumor selective apoptotic cell death therapy. However, apoptosis resistance is often encountered. The main aim of this study was to investigate the apoptotic mechanism underlying TRAIL sensitivity in three bortezomib (BTZ)-resistant NSCLC variants, combining induction of both the intrinsic and extrinsic pathways.

**Methods:** Sensitivity to TRAIL in BTZ-resistant variants was determined using a tetrazolium (MTT) and a clonogenic assay. A RT-qPCR profiling mRNA array was used to determine apoptosis pathway-specific gene expression. The expression of these proteins was determined through ELISA assays and western Blotting, while apoptosis (sub-G1) and cytokine expression were determined using flow cytometry. Apoptotic genes were silenced by specific siRNAs. Lipid rafts were isolated with fractional ultracentrifugation.

**Results:** A549BTZR (BTZ-resistant) cells were sensitive to TRAIL in contrast to parental A549 cells, which are resistant to TRAIL. TRAIL-sensitive H460 cells remained equally sensitive for TRAIL as H460BTZR. In A549BTZR cells, we identified an increased mRNA expression of *TNFRSF11B* [osteoprotegerin (OPG)] and caspase-1, -4 and -5 mRNAs involved in cytokine activation and immunogenic cell death. Although the OPG, interleukin-6 (IL-6), and interleukin-8 (IL-8) protein levels were markedly enhanced (122-, 103-, and 11-fold, respectively) in the A549BTZR cells, this was not sufficient to trigger TRAIL-induced apoptosis in the parental A549 cells. Regarding the extrinsic apoptotic pathway, the A549BTZR cells showed TRAIL-R1-dependent TRAIL sensitivity. The shift of TRAIL-R1 from non-lipid into lipid rafts enhanced TRAIL-induced apoptosis. In the intrinsic apoptotic pathway, a strong increase in the mRNA and protein levels of the anti-apoptotic myeloid leukemia cell differentiation protein (Mcl-1) and B-cell leukemia/lymphoma 2 (Bcl-2) was found, whereas the B-cell lymphoma-extra large (Bcl-xL) expression was reduced. However, the stable overexpression of Bcl-xL in the A549BTZR cells did not reverse the TRAIL sensitivity in the A549BTZR cells, but silencing of the BH3 Interacting Domain Death Agonist (BID) protein demonstrated the importance of the intrinsic apoptotic pathway, regardless of Bcl-xL.

**Conclusion:** In summary, increased sensitivity to TRAIL-R1 seems predominantly related to the relocalization into lipid rafts and increased extrinsic and intrinsic apoptotic pathways.

## INTRODUCTION

Non-small cell lung cancer (NSCLC) constitutes approximately 85% of all lung cancer cases, and it is associated with a very poor 5-year survival rate (approximately 16%)^[1-3]^. Although immunotherapy has shown considerable benefits for a subpopulation of NSCLC patients^[4,5]^, there is still a need for novel targeted agents, including those targeting apoptosis. Apoptosis is required for the depletion of redundant, damaged, or transformed cells, such as viral-infected or malignant cells. However, in most tumor cells, the ability to activate apoptosis is inhibited due to the overexpression of pro-survival proteins and/or loss of proapoptotic proteins^[6,7]^. Therefore, the specific activation of apoptosis in tumor cells is a potent strategy to treat cancer. Currently, several tumor necrosis factor (TNF)-related apoptosis-inducing ligand (TRAIL)-based drugs have been or are being investigated for proapoptotic activity in clinical phase II and III trials in NSCLC. One of the major aims of these trials is to test the induction of the immune response against the tumor by TRAIL, although the main problems of TRAIL-based therapeutics are resistance, poor pharmacokinetics, and short half-life^[8,9]^. Although apoptosis is an immunologically silent process, there are several links combining it with immune response, e.g., caspase-8^[10,11]^. In addition, chemo- and radiotherapy, as well as growth factor receptor inhibitors targeting epidermal growth factor receptor (EGFR), anaplastic lymphoma kinase-echinoderm microtubule-associated protein-like 4 fusion (ALK-EML), and neurotrophic tyrosine receptor kinase (NTRK), are extensively being used for the treatment of NSCLC, and these treatments also induce apoptosis. Thus, the current treatment modalities trigger at least the apoptotic cell death pathway. Therefore, more insight into the resistance mechanism of apoptosis induction will help to improve the current therapy^[10]^.

The initiation of apoptosis can be regulated via extrinsic and intrinsic apoptotic pathways, which will ultimately result in the activation of the effector caspases^[10]^. The activation of the extrinsic apoptotic pathway is achieved by the binding of TNF family members to their associated receptors. Several variants of TRAIL have been or are being investigated for their clinical benefit in phase I, II and III clinical trials (clinicaltrials.gov^[12-15]^). TRAIL agonists can bind to the death receptors TRAIL-R1 and TRAIL-R2 and the decoy receptors TRAIL-R3 and TRAIL-R4. The activation of TRAIL-R1 and TRAIL-R2 results in the trimerization of the receptors and the formation of the intracellular death-inducing signaling complex (DISC) involving Fas-associated death domain (FADD) and caspase-8. This will lead to the activation of caspase-8, which, in turn, can activate effector caspase-3 directly or, alternatively, via the cleavage of BH3 Interacting Domain Death Agonist (BID) into a truncated form, tBID. Truncated BID facilitates the localization of Bak and Bax into the mitochondrial membrane, thereby initiating the intrinsic apoptotic pathway, resulting in the release of cytochrome C and SMAC/DIABLO. Cytochrome C is required for the formation of the apoptosome, where caspase-9 will be activated, leading to the activation of caspase-3, resulting in apoptosis^[16-18]^. Apart from the primary DISC, a secondary signaling complex can be formed containing receptor-interacting protein kinase 1 (RIPK1) and TNF receptor-associated factor 2 (TRAF2). This secondary complex can activate several protein kinase pathways that promote cell proliferation, including the activation of nuclear factor kappa-light-chain-enhancer of activated B cells (NF-κB), which can enhance the expression of a number of anti-apoptotic proteins, such as cellular FLICE-like inhibitory protein (c-FLIP) and X-linked inhibitor of apoptosis protein (XIAP)^[19,20]^.

Furthermore, the distribution of the TRAIL receptors within lipid rafts forms an activated DISC and induces cell death, whereas localization in the non-lipid rafts recruits the secondary complex, thereby initiating cell survival^[21-24]^. More recently, β-catenin was found to induce the redistribution of the cell surface expression of TRAIL-R1 and -R2, leading to increased caspase-3/8 activation and apoptosis, thus enhancing TRAIL sensitivity^[25]^. Other features that contribute to TRAIL resistance consist of mutations in the receptors^[26,27]^ or O-glycosylation of the intracellular part of the receptor^[28]^. In addition, high levels of c-FLIP^[29]^ and phosphoprotein enriched in diabetes (PED)^[30]^ have been described to interfere with the formation of DISC, thereby hampering the apoptotic pathway. Further downstream in the apoptotic pathway, resistance can be dependent on high expression levels of the anti-apoptotic B-cell leukemia/lymphoma 2 (Bcl-2) family proteins Bcl-2, B-cell lymphoma-extra large (Bcl-xL), and myeloid leukemia cell differentiation protein (Mcl-1), which are key regulators of the mitochondrial-dependent apoptotic pathway^[31,32]^.

To increase the therapeutic potential of TRAIL, a combination of various therapeutic agents has been examined, including bortezomib (BTZ). BTZ is a prototype proteasome inhibitor that primarily targets the β5 subunit of the proteasome, thereby inhibiting the degradation of many proteins^[33,34]^. Initially, the rationale for combining TRAIL with BTZ was based on the inhibitory effect of BTZ on IκB degradation, consequently repressing NF-κB activity^[35]^. However, over the years, BTZ has been recognized to trigger a multitude of antiproliferative effects, all of which could contribute to increased TRAIL sensitization^[36]^, such as additional inhibition of the intrinsic apoptotic pathway.

In a previous study, we examined the mechanisms of acquired BTZ resistance in several NSCLC cell lines. The enhanced expression levels of the β-subunits of the proteasome, as well as point mutations, such as *Ala49Thr*, *Met45Val*, and *Cys52Phe* substitutions, within the β5 subunit of the proteasome, were shown to contribute to BTZ resistance^[37]^. In the present study, we examined the effect of TRAIL on these BTZ-resistant NSCLC cells. Interestingly, we found markedly enhanced TRAIL sensitivity in the BTZ-resistant A549 cells, whereas the parental cells were TRAIL-resistant. Both cell lines were used as a model to further investigate the molecular mechanism underlying TRAIL resistance.

## METHODS

### Cell culture and transfection

The human NSCLC H460, A549, and SW1573 cell lines were obtained from the American Type Culture Collection (Manassas, VA, USA). H460 and A549 were grown in RPMI-1640 and SW1573 in DMEM (Lonza, Verviers, Belgium) supplemented with 10% fetal bovine serum (Greiner Bio-One, Frinckenhausen, Germany) and 100 units/mL of penicillin/streptomycin (Lonza, Verviers, Belgium). The cells were grown at 37 °C in a humidified atmosphere of 5% CO_2_ and regularly checked for mycoplasma infections.

BTZ-resistant cells were established by exposing cells to gradually increasing concentrations of BTZ over a period of at least 6 months: H460 (from 5 to 100 nM), A549 (5-40 nM) and SW1573 (5-50 nM). The resistant variants are further referred to as H460BTZR, A549BTZR, and SW1573BTZR, respectively^[37]^. BTZ-resistant cells were cultured in BTZ-free medium for at least 72 h before the initiation of the experiments to exclude the interference of the selective BTZ concentrations.

For the generation of stable transfectants, H460, A549, and A549BTZR cells were transfected with 10 µg cDNA encoding Bcl-xL subcloned into the expression vector pEFLAGpGKpuro^[38]^ using FuGENE® 6 Transfection Reagent (Promega, Madison, WI, USA) according to the manufacturer’s protocol. Selection was made using increasing concentrations of puromycin (Sigma-Aldrich, Zwijndrecht, the Netherlands) ranging from 1-2 µg/mL.

Drug sensitivity was determined using the tetrazolium (MTT) assay as described earlier^[39]^. Briefly, cells were plated in 96-wells plates and 24 h (day 0) after attachment drugs (BTZ or TRAIL) were added. After 72 h (day 3), the growth inhibition was measured by using the MTT assay as described. IC50 values were determined by correcting optical density at day 3, by the initial optical density (OD) on the day of drug addition (day 0). The value on day 3 was set at 100% and that on day 0 at 0%. IC50 values were defined as the concentration at which 50% growth inhibition was established. OD values on day 3 below that of day 0 are defined as cell kill.

### Reagents and antibodies

BTZ (Velcade®) was obtained from Millennium Pharmaceuticals Inc. (Cambridge, MA, USA). RhTRAIL^[39]^, TRAIL-R1-specific TRAIL variant 4C7^[40]^, and TRAIL-R2-specific TRAIL variant D269H/E195R^[41]^ were produced non-commercially in cooperation with IQ-Corporation (Groningen, the Netherlands) following a protocol described earlier. The broad-spectrum caspase inhibitor zVAD-fmk was purchased from Promega, Madison, USA. For the western blot, the primary antibodies included mouse monoclonal caspase-8 (1C12), rabbit anti-caspase-9 (human specific), rabbit anti-cleaved caspase-9 (D330) (human specific), rabbit anti-caspase-3, rabbit anti-cleaved caspase-3 (Asp175), rabbit anti-PARP, rabbit anti-FLIP, rabbit anti-Bcl-2, rabbit anti-Bcl-xL, rabbit anti-Mcl-1, rabbit anti-BID, anti-caspase-1, anti-caspase-4, anti-caspase-5 (Cell Signaling Technology, Danvers, MA, USA), mouse anti-XIAP clone 2F1 (MBL International, Woburn, MA, USA), mouse monoclonal anti-NOXA, rabbit anti-TRAIL receptor 2 (Merck KGaA, Darmstadt, Germany), goat anti-DR4 (C20) (Santa Cruz Biotechnology, Heidelberg, Germany), and anti-β-actin (Sigma-Aldrich Chemicals, Zwijndrecht, the Netherlands), which were diluted in InfraRedDye blocking buffer (Rockland Inc., Pottstown, PA, USA). The secondary antibodies were goat-a-mouse-InfraRedDye (1:10,000, 800CW;#926-32210 and 680;#926-32220, Westburg, Leusden, the Netherlands), goat-a-rabbit-InfraRedDye (800CW;926-32211 and 680;#926-32221, Westburg, Leusden, the Netherlands), or donkey-a-goat-InfraRedDye (800CW; #926-32214, Westburg, Leusden, the Netherlands). Necrostatin-1, a specific RIPK1 inhibitor of necroptosis^[42]^, was obtained from Selleck (Houston, TX, USA).

### Clonogenic survival

Clonogenic survival was performed as described previously^[43]^. Briefly, cells were seeded at a density of 250 cells/well and treated with 100 ng/mL TRAIL. After 6 h exposure, the cells were washed and the medium replaced by drug-free medium, followed by 1-2 weeks incubation to allow for colony formation. At this stage, the cells were washed with PBS, fixed with 99% ethanol, and washed again. The colonies were stained with 10% Giemsa (Merck, Darmstadt, Germany), and those consisting of >50 cells were counted. The surviving fraction was determined by dividing the amount of colonies by the amount of cells plated. The surviving fraction of untreated cells was set to 1.

### Western blot analysis

Western blot analysis was performed as described previously^[37]^. Briefly, the protein samples were separated by 8%-15% SDS PAGE, electroblotted onto a PVDF membrane (Millipore, Amsterdam, the Netherlands) and blocked in InfraRedDye blocking buffer. The membrane was probed with the indicated primary antibodies (overnight at 4 °C), followed by 1 h incubation with the indicated secondary antibodies. Fluorescent proteins were detected by an Odyssey Infrared Imager (LI-COR Biosciences, Lincoln, NE, USA), 84 µm resolution, 0 mm offset, and with high quality.

### Cell surface expression of TRAIL receptors

TRAIL receptor membrane levels were determined on BTZ-resistant cells and their parental counterparts, as described previously^[44]^. Briefly, cells were collected, washed, and incubated with primary antibodies against TRAIL-R1, TRAIL-R2, TRAIL-R3, and TRAIL-R4 (clones HS101, HS201, HS301, and HS402, 10 µg/mL; Alexis, London, UK) for 15 min at room temperature (RT). Mouse IgG1 was used as a negative control. The cells were washed, followed by incubation with rabbit-anti-mouse PE labeled (DAKO) for 15 min at RT. The cells were washed, and the fluorescence was measured on a FACS-Calibur flow cytometer. The relative fluorescence intensity (RFI) was calculated as the mean fluorescence intensity (MFI) of the TRAIL receptor-MFI of IgG1. The basal levels were set at 100%.

### RNA interference

For the transient gene knockdown, cells were seeded at 2.5 × 10^5^ per well in a 6-well tissue culture plate and allowed to settle overnight. SiRNA duplexes were formed using oligofectamine reagent according to the manufacturer’s instructions (Invitrogen BV). The culture medium was replaced with OPTI-MEM®I (Invitrogen, Breda, the Netherlands), and the cells were transfected with 100 nM small interfering RNA (siRNA) molecules targeting the sense GGAAGAGAACAGGACUGAGGC and antisense GCCUCAGUCCUGUUCUCUUCC strands of *Bcl-xL* and the GAAUAGAGGCAGAUUCUGA sense and UCAGAAUCUGCCUCUAUUC antisense strands for *BID* (Eurogentech, Seraing, Belgium). As a control, siRNA with no homology to the human genome was used. After 24 h, the cells were reseeded for flow cytometric analysis.

### Flow cytometric analysis of cell cycle distribution

Cell cycle analysis and cell death measurements were performed as described previously^[45]^. Briefly, cells were harvested after 24 h of exposure to TRAIL and centrifuged for 5 min at 1,200 rpm (250 *g*). Subsequently, the cells were stained with propidium iodide buffer (0.1 mg/mL propidium iodide, 0.1% RNase A) in the dark on ice. The DNA content was analyzed by fluorescence-activated cell sorting (FACS) analysis (Becton Dickinson, Immunocytometry Systems, San Jose, CA, USA) with an acquisition of 10,000 events. Cell death was determined by the sub-G_1_ peak.

### Fractionation lipid rafts

Fractionation of the lipid rafts from the non-lipid rafts was performed as described previously^[23]^. Briefly, 1 × 10^8^ cells were lysed on ice in 2 mL MNX buffer [1% Triton X-100 in 25 mM MES and 150 mM NaCl (pH 6.5)] supplemented with 1 mM phenylmethylsulfonyl fluoride and protease inhibitor cocktail (Sigma-Aldrich Chemicals, Zwijndrecht, the Netherlands) and then homogenized. The homogenates were resuspended in 2 mL 90% sucrose dissolved in MNX buffer and gently overlaid with 4 mL 35% sucrose and 4 mL 5% sucrose. The samples were centrifuged at 175,000× *g* in a SW32Ti rotor with an Optima L-80 XP centrifuge (Beckman, Brea, CA, USA) for 16 h at 4 °C. Ten fractions of 1 mL were collected from top to bottom of the gradient and analyzed by western blot.

### mRNA expression of apoptosis specific genes by RT-qPCR

To detect alterations in the mRNA expression levels of several pro- and anti-apoptotic genes, we performed an RT^2^ Profiler PCR Array (SABionsciences, Frederick, MD, USA), which was capable of detecting 84 apoptotic genes with qPCR according to the manufacturer’s instructions. Briefly, the RNA was isolated using the RNeasy 96 kit (Qiagen Inc., Valencia, CA, USA). The cDNA was prepared with the RT^2^ First strand kit, mixed with the RT^2^SYBR Green qPCR master mix, and aliquoted across the PCR array. Amplification data (C_t_ values) were collected with an ABI 7500 RT-PCR system. ΔΔC_t_ was determined as (A549BTZRC_t_ - A549BTZRC_t housekeeping genes_) - (A549C_t_ - A549C_t housekeeping genes_). The fold change was calculated by 2^ΔΔCt^, indicating the upregulation of genes with a fold change > 1 in the A549BTZR cells and the downregulation of genes with a fold change < 1.

### Detection of human osteoprotegerin by ELISA

The levels of secreted osteoprotegerin (OPG) were detected using a RayBio® Human Osteoprotegerin ELISA Kit according to the manufacturer’s instructions (RayBiotech, Inc., Norcross, GA, USA). Briefly, supernatants of different cell lines were collected, centrifuged for 5 min at 1,500 rpm (300× *g*) and sampled on an osteoprotegerin microplate coated with anti-human osteoprotegerin. After incubation for 2.5 h at RT, the wells were washed and incubated with biotinylated antibody for 1 h at RT. The solution was discarded, and after washing, streptavidin solution was added for 45 min at RT. After washing, TMB One-Step Substrate Reagent was added for 30 min at RT. Stop solution was added, and the optical density was measured at 450 nm.

### Secretion of cytokines

A549 and A549BTZR cells were plated in 6-well plates at a density of 3 × 10^5^ cells. After 24 h, the supernatant was collected and centrifuged for 5 min at 1,500 rpm to remove detached cells. According to the manufacturer’s protocol, the cytometric bead array (CBA) human inflammatory cytokines kit (BD biosciences, San Jose, CA, USA) was used for the quantitative measurement of interleukin-8 (IL-8), interleukin-1β (IL-1β), interleukin-6 (IL-6), interleukin-10 (IL-10), TNF, and interleukin-12p70 (IL-12p70). Measurements were performed on a FACS-Calibur flow cytometer (Becton Dickinson, Immunocytometry Systems, San Jose, CA, USA) using the CellQuest program.

### Statistics

Statistical evaluation was performed using the Student t-test, with *P* < 0.05 as the level of significance, unless otherwise indicated.

## RESULTS

### Phenotypic alteration for TRAIL sensitivity in A549BTZR cells

**Figure 1:**
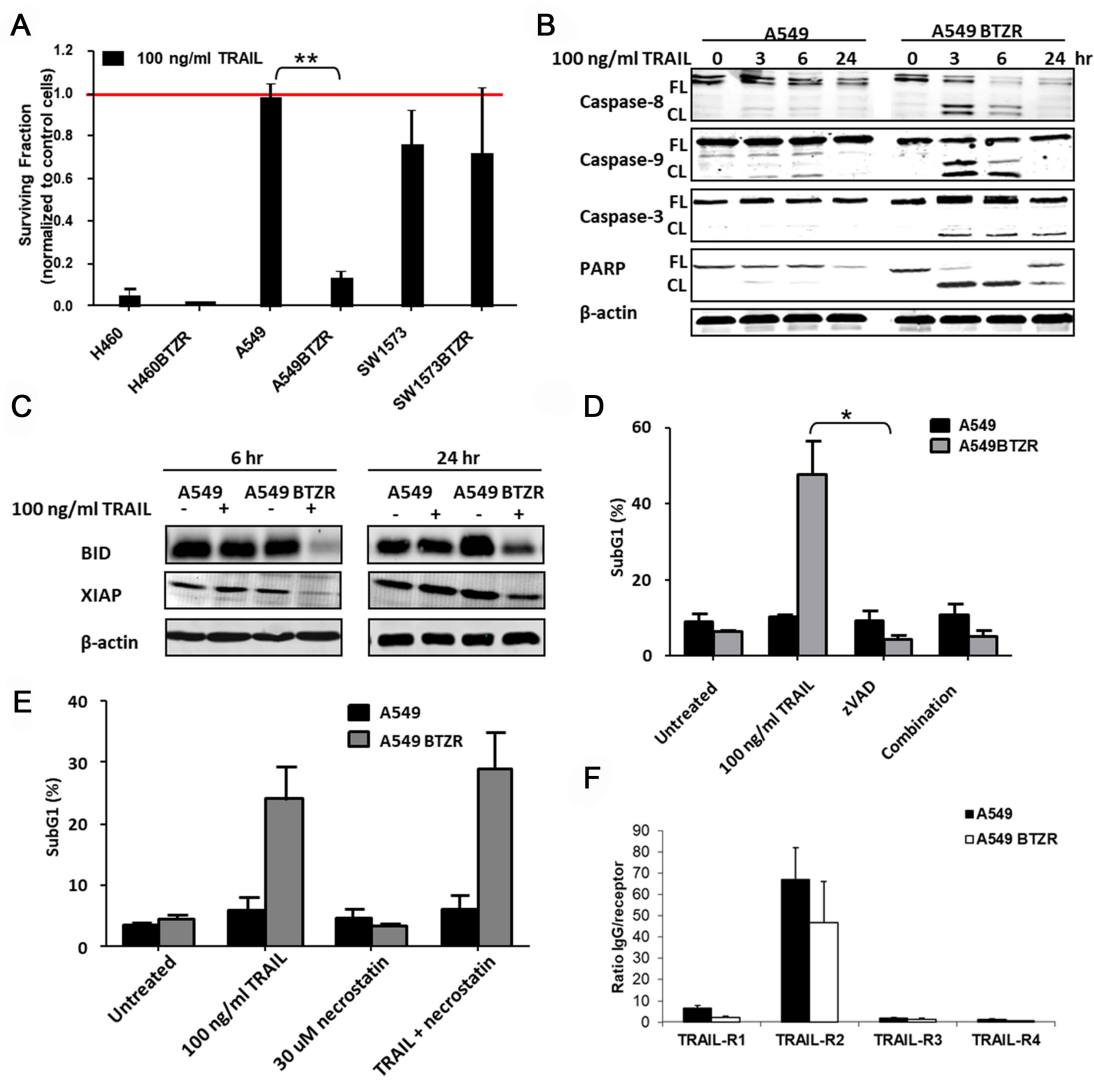
Acquired BTZ-resistant A549 cells demonstrate reversed phenotypic TRAIL sensitivity. (A) The clonogenic outgrowth was significantly reduced in both the H460BTZR (not measurable) and A549BTZR cells after exposure to 100 ng/mL TRAIL for 6 h; (B) Western blot analysis of caspase and PARP cleavage shows the enhanced cleavage already after 6 h in the A549BTZR cells; (C) BID and XIAP were reduced in the A549BTZR cells after exposure to 100 ng/mL TRAIL for 6 and 24 h; (D) the ZVAD (100 µg/mL) completely reduced the TRAIL-enhanced apoptosis after 24 h exposure as determined with Flow Cytometry; (E) Incubation with 30 μM necrostatin for 24 h did not result in reduced TRAIL-induced apoptosis as determined with Flow Cytometry; (F) Expression of TRAIL in A549BTZR cells as determined with Flow cytometry. Similarly, the TRAIL expression was not different between parental and resistant variants of H460 and SW1573 cells. The values are the means ± SEM and the western blots depicted represent at least three independent experiments. Similarly, the effect of TRAIL was significantly higher in A549BTZR compared to A549 cells. ^**^P < 0.001; ^*^P < 0.01. BTZ: Bortezomib; TRAIL: TNF-related apoptosis-inducing ligand; TNF: tumor necrosis factor; BID: BH3 Interacting Domain Death Agonist; XIAP: X-linked inhibitor of apoptosis protein.

Previously, we generated acquired BTZ resistance in a panel of NSCLC cells that displayed different TRAIL sensitivities^[18,37]^. We used H460 (IC_50_: 6 ng/mL TRAIL), A549, and SW1573 cells (IC_50_: > 200 ng/mL TRAIL). Since BTZ is a potent sensitizer for TRAIL-induced apoptosis, we examined TRAIL sensitivity in the generated acquired BTZ-resistant cell lines. Using the MTT assay for determination of initial sensitivity, the IC50 for TRAIL in H460BTZR cells slightly increased to 9.9 ng/ml, did not change in SWS1573BTZR cells (> 200 ng/mL), but decreased in A549BTZR cells (11.3 ng/mL). However, at a higher concentration (100 ng/mL), we found that in A549BTZR and H460BTZR cells, a higher extent of cell kill was observed compared to A549 and H460 cells. Based on the MTT assay data, we chose one concentration of TRAIL (100 ng/mL) to be tested in clonogenic assays since this assay gives a better estimate of surviving fractions. In clonogenic assays, TRAIL sensitivity was slightly increased in H460BTZR compared to parental H460 cells [Figure 1A]. Interestingly, also with the clonogenic assay, the A549BTZR cells were significantly more sensitive to TRAIL-induced apoptosis, as shown by the strong decline in the clonogenic outgrowth with surviving fractions of 0.98 to 0.13 for the A549 and A549BTZR cells, respectively (*P* < 0.005). Therefore, for further mechanistic studies, we focused on the A549 cells and their BTZ-resistant variant A549 BTZR.

In order to determine whether increased apoptosis was responsible for this collateral sensitivity, we analyzed time-dependent caspase cleavage after exposure to TRAIL using western blot analysis. TRAIL increased levels of caspase-8, caspase-9, caspase-3, and PARP cleavage in the A549BTZR cells compared to the parental cells [Figure 1B]. In addition, both BID and XIAP were cleaved after 6 h of exposure to TRAIL in the A549BTZR cells, whereas no cleavage was detected in the A549 cells up to 24 h of treatment [Figure 1C]. To determine whether the increased TRAIL-induced cell death was caspase-dependent, we investigated the effect of zVAD, a broad-spectrum caspase inhibitor, on TRAIL-induced apoptosis [Figure 1D]. After 24 h of exposure, the cell death was significantly reduced (*P* < 0.01) when exposed to both TRAIL and zVAD, indicating that the TRAIL-induced apoptosis in the A549BTZR cells was caspase-dependent. The exposure of cells to 30 µM necrostatin in combination with TRAIL did not reduce the amount of cell death in A549BTZR, thereby excluding the activation of cell death via necroptosis [Figure 1E]. TRAIL expression was similar between parental and resistant cells [Figure 1F].

### mRNA and protein levels of apoptotic proteins are differentially expressed in A549BTZR cells

**Figure 2:**
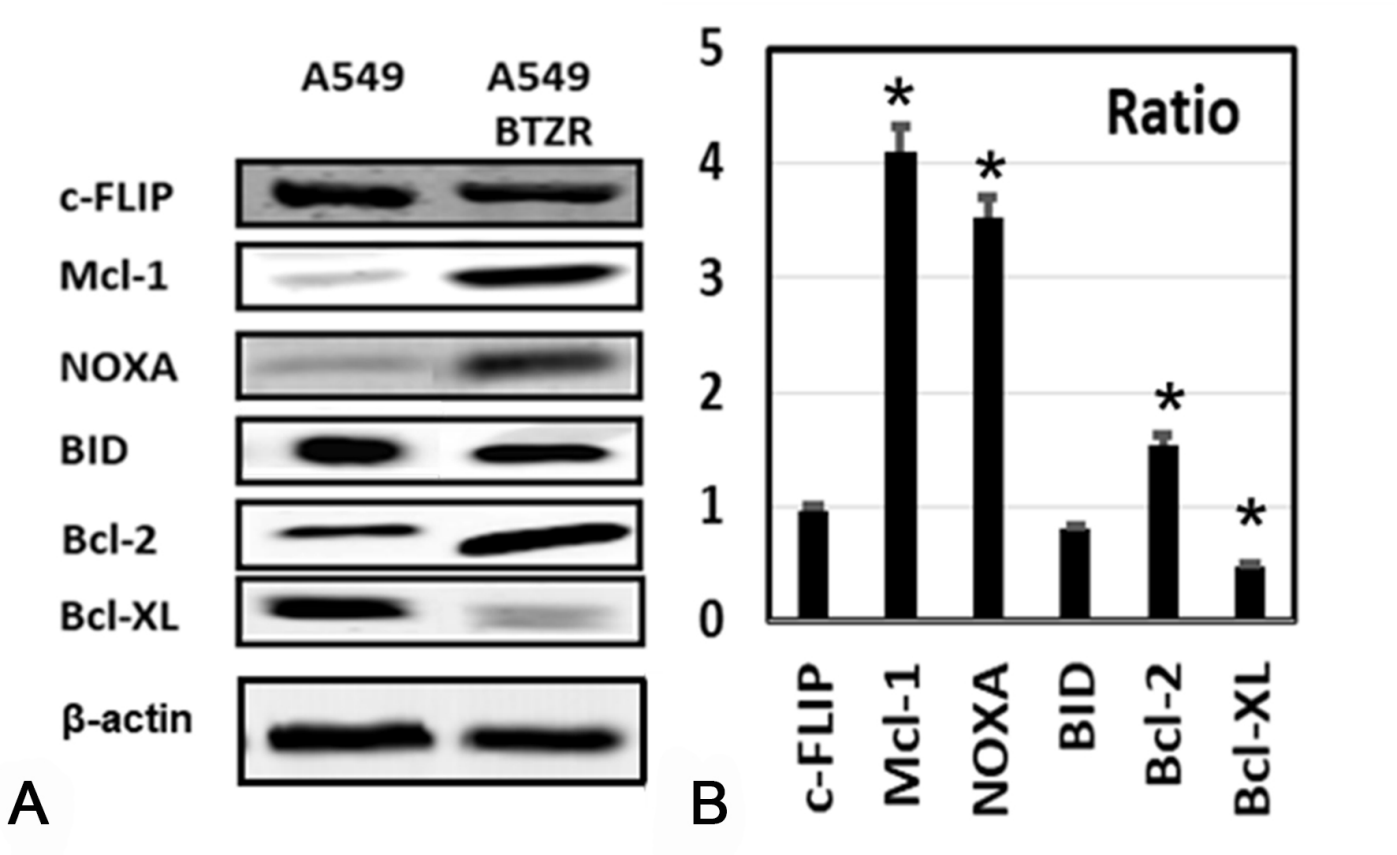
Basal protein levels were differently expressed in the A549 and A549BTZR cells. (A) Western blot analysis was used to detect the basal expression levels of several proteins in the A549 and A549BTZR cells. The blots are representative of at least three separate experiments. β-actin was used as a loading control; (B) Scans showing the ratio A549BTZ/A549. ^*^P < 0.01.

The enhanced TRAIL sensitivity in the A549 BTZ-resistant variant provides a very interesting model to further characterize the underlying mechanisms that regulate TRAIL-induced apoptosis. Therefore, we determined the basal mRNA expression levels of 84 apoptosis-related genes in A549 and A549BTZR cells using an RT^2^Proflier^TM^ PCR array. The top ten genes that were overexpressed or downregulated in A549BTZR are shown in Table 1. In line with the alterations found with the RT^2^Proflier^TM^ PCR array, we observed that the protein expression levels of c-FLIP and BID were not differentially expressed in A549 and the BTZ-resistant counterpart [Figure 2]. Moreover, the expression level of Bcl-xL was strongly reduced in the A549BTZR cells, whereas the expression of Bcl-2 and Mcl-1 increased. Although Phorbol-12-myristate-13-acetate-induced protein 1 (NOXA) was not included in the RT-PCR array, we also evaluated the protein levels of this Mcl-1 inhibitor and found that NOXA was also overexpressed in the A549BTZR cells. The relationships among the downregulated and upregulated apoptotic genes were further analyzed by an IPA pathway analysis [Table 2], and schematic figures of the network functions are represented in Supplementary Figure 1. Cell death, cell-mediated immune response, and cellular development were predominantly found as associated network functions. Taken together, these findings indicate that a broad range of pathways relevant to cell survival are altered in A549BTZR cells.

**Table 1:** A549BTZR cells display an altered mRNA expression pattern

	**Fold Change**
**A: Gene**	
*TNFRSF11B*	75
*CASP5*	28
*CASP4*	21
*CASP1*	10
*CD70*	8
*MCL1*	4
*BCL2*	4
*TNFRSF25*	3
*CASP3*	3
*GADD45A*	2
**B: Gene**	
*DAPK1*	0.0006
*PYCARD*	0.0206
*TNFSF8*	0.0304
*BCL2A1*	0.0311
*TNFRSF9*	0.0404
*TNFRSF21*	0.0519
*BCL2L1*	0.0644
*TP53*	0.0850
*BAD*	0.1325
*BFAR*	0.1554

RT-qPCR analysis resulted in a top 10 of mRNAs that were expressed higher (A) or lower (B) in A549BTZR cells. Values were corrected for the average of five different housekeeping genes. Values found for A549 cells were set to 1 and values of A549BTZR cells were correlated to determine the fold change.

**Table 2:** Network functions associated with the altered mRNA expression levels in the A549BTZR cells

**ID**	**Associated network functions**	**Score**
1	Cell death, embryonic development, renal necrosis/cell death	49
2	Cell death, cell-mediated immune response, cellular development	41
3	Cell death, cell-mediated immune response, cellular development	32
4	Cell death, dermatological diseases and conditions, genetic disorder	15
5	Cell death, cell cycle, antimicrobial response	13

An analysis using IPA resulted in a list of the top 5 networks associated with the modulated mRNA expression levels in the A549BTZR cells compared to the A549 cells.

### A549BTZR cells secrete high levels of osteoprotegerin

**Figure 3:**
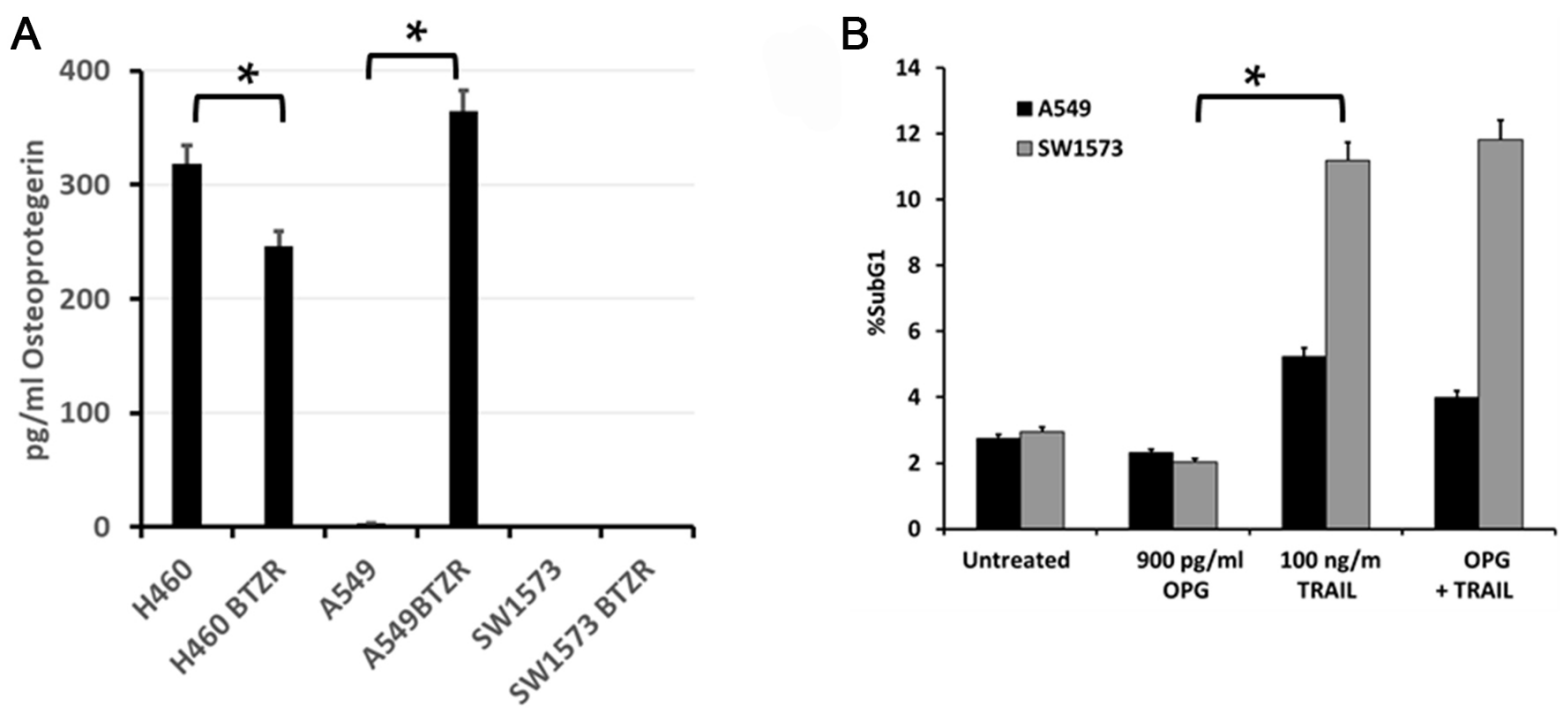
Enhanced secretion of OPG by A549BTZR did not induce TRAIL sensitivity. (A) With the use of ELISA, the secreted OPG levels were measured in the supernatant of the different cell lines; ^*^significantly different (*P* < 0.01). The secretion of OPG by both SW1573 variants was 0.41 pg/mL; (B) Cell death was measured in the TRAIL-resistant A549 and SW1573 cells after incubation with 900 pg/mL OPG for 4 h followed by the addition of 100 ng/mL TRAIL for 24 h. The effect of TRAIL was significantly higher than that of OPG (^*^P < 0.01). The values are the means of at least three independent experiments ± SEM. OPG: Osteoprotegerin; TRAIL: TNF-related apoptosis-inducing ligand; TNF: tumor necrosis factor.

The *TNFRSF11B* mRNA, encoding the soluble TRAIL receptor, OPG, was the highest upregulated gene in A549BTZR cells. This finding is remarkable since OPG is thought to inhibit the apoptosis-inducing effect of TRAIL. An ELISA analysis showed that the secretion of OPG in the A549BTZR cells strongly increased by 122-fold, from 3 pg/mL to 364 pg/mL OPG for the A549 and A549BTZR cells, respectively [Figure 3A]. Notably, the TRAIL-sensitive H460 cells also had a high level of OPG secretion, but were very low in both SW1573 and SW1573BTZR cells. However, the incubation of the TRAIL-resistant A549 or SW1573 cells with 900 pg/mL OPG for 4 h prior to the TRAIL treatment did not affect cell death activation [Figure 3B]. Thus, the increases in OPG secretion could not be linked with the increased TRAIL sensitivity in the A549BTZR cells.

### Altered secretion of cytokines does not influence TRAIL sensitivity

**Figure 4:**
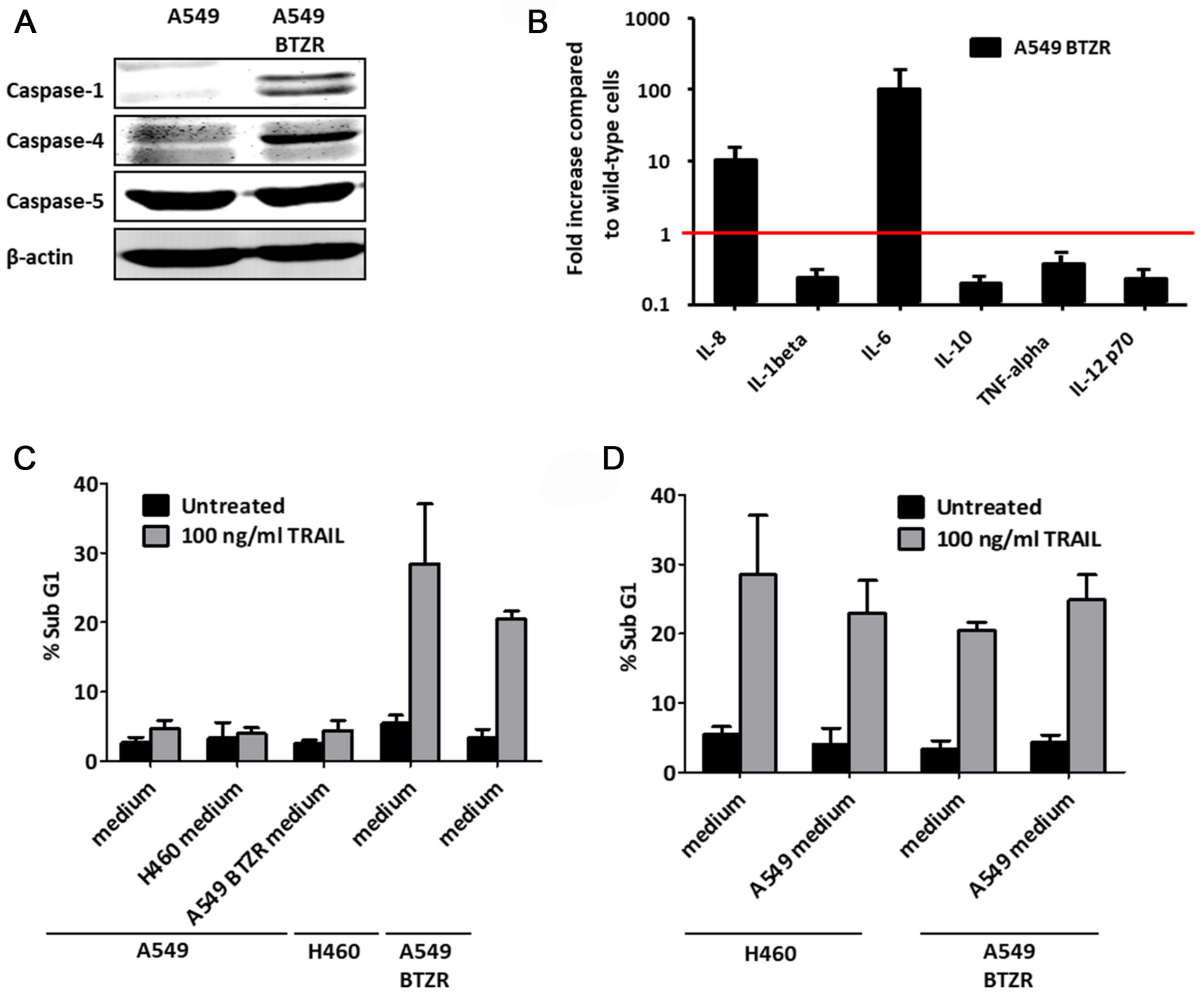
Different secretion products from the A549BTZR cells did not trigger TRAIL sensitivity. (A) Western blot analysis was used to detect the basal expression levels of caspase-1, -4 and -5 in A549 and A549BTZR cells; (B) Supernatant of the A549 and A549BTZR cells was used to determine the cytokine secretion. The cytokine secretion levels detected in the A549BTZR supernatant were compared with the secretion by A549 cells, which was set to 1 (calculated from the means); (C) Cell death (subG1) was measured in A549 cells exposed to 100 ng/mL TRAIL for 24 h in supernatant collected from the H460 and A549BTZR cells. As a control, H460 and A549BTZR cells were exposed to 100 ng/mL TRAIL for 24 h in fresh medium; (D) Cell death (SubG1) in the H460 and SW1573 cells was determined after exposure to 100 ng/mL TRAIL for 24 h in culture medium or supernatant collected from A549 cells. The values are the means ± SEM of at least three independent experiments, while the blots are representative of at least 3 separate experiments. The effect of TRAIL treatment on H460 and A549BTZR cells on induction of apoptosis (C and D) was significant (*P* < 0.001). TRAIL: TNF-related apoptosis-inducing ligand; TNF: tumor necrosis factor.

The mRNAs for *caspase*-1, *caspase*-4, and *caspase*-5 also strongly increased in the BTZ-resistant cells [Table 1]. These caspases are involved in the regulation of inflammatory responses [Supplementary Figure 1]^[46]^. A western blot analysis showed an increased expression of caspase-1 and caspase-4 but not of caspase-5 [Figure 4A]. Since these caspases are part of the inflammasome and play a role in the immune system by promoting the maturation and secretion of pro-inflammatory cytokines, we examined the cytokine secretion of A549 and A549BTZR cells. IL-8 and IL-6 levels were found to be increased by 11- and 103-fold, respectively, whereas the secretion of IL-1β, IL-10, TNF-α, and IL-12 p70 was reduced [Figure 4B].

Since several alterations in cytokine secretion were found between TRAIL-sensitive and -resistant cells, we investigated whether TRAIL sensitivity is affected by a combination of secreted factors and not just by one factor such as OPG. Therefore, we cultured A549 cells in conditioned medium collected from TRAIL-sensitive H460 or A549BTZR cells and exposed the cells to 100 ng/mL TRAIL. However, the apoptosis-inducing effect of TRAIL did not change [Figure 4C]. The opposite was also examined by incubating H460 and A549BTZR cells with A549-conditioned medium with or without 100 ng/mL TRAIL. However, the conditioned A549 medium also did not affect TRAIL sensitivity in H460 and A549BTZR cells [Figure 4D]. Taken together, these results indicate that although the secretion of OPG and other cytokines is different in TRAIL-sensitive A549BTZR cells compared to parental cells, this does not affect TRAIL sensitivity.

### TRAIL-R1 mediates TRAIL-induced apoptosis in A549 BTZ-resistant cells, and its localization is altered to the lipid rafts

**Figure 5:**
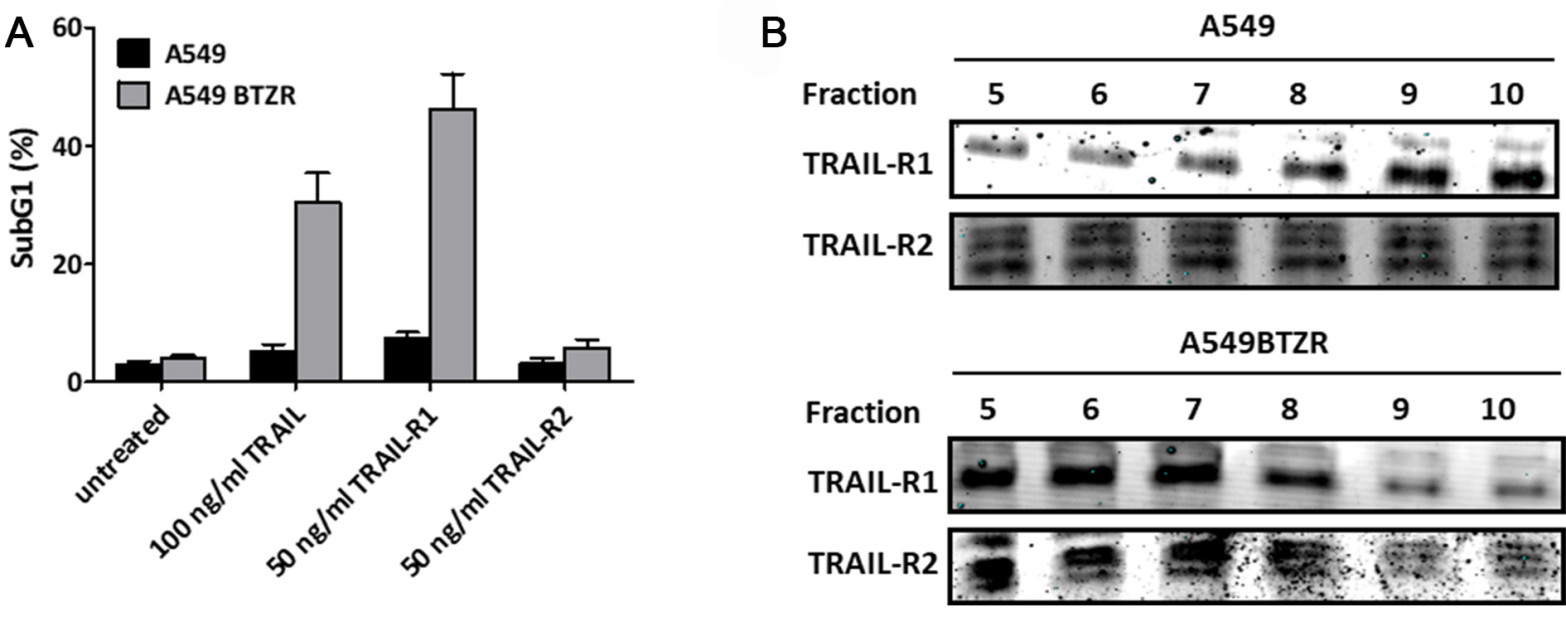
A549BTZR TRAIL-induced apoptosis was mediated via TRAIL-R1, which was redistributed into lipid rafts. (A) Cell death was determined in the A549 and A549BTZR cells after exposure to 100 ng/mL TRAIL, 50 ng/mL 4C7 (TRAIL-R1-specific TRAIL variant), or 50 ng/mL D269HE195R (TRAIL-R2-specific TRAIL variant) for 24 h. The effect of TRAIL and TRAIL-R1 was significant in A549BTZR cells compared to A549 parental (*P* < 0.0001); (B) Western blot analysis of the TRAIL-R1 and -R2 expression levels in different cellular fractions, with fractions 5-7 and fractions 8-10 representing the lipid and non-lipid rafts, respectively. The values are the means ± SEM of at least three independent experiments, while blots are also representative of at least 3 separate experiments. TRAIL: TNF-related apoptosis-inducing ligand; TNF: tumor necrosis factor.

To further examine the underlying mechanism of TRAIL sensitization, we determined which of the TRAIL receptors mediates TRAIL-induced apoptosis. A549BTZR and A549 cells were exposed to TRAIL-receptor-targeting TRAIL-variants-targeting antibodies that were either specific for TRAIL-R1 (4C7) or TRAIL-R2 (D269H/E195R). Strong induction of cell death was detected when the A549BTZR cells were exposed to 50 ng/mL TRAIL-R1 antibody, whereas no cell death was found after exposure to the TRAIL-R2-specific antibody [Figure 5A], indicating a pivotal role for TRAIL-R1. TRAIL receptor levels are often upregulated upon BTZ treatment as a mechanism to sensitize cells for TRAIL-induced apoptosis^[6]^. However, the mRNA levels were not significantly altered in the A549BTZR cells. In line with this, a FACS analysis showed no significant difference in the TRAIL receptor expression levels at the cell membrane between the parental A549 and A549BTZR cells [Figure 5B]. Though the TRAIL receptor expression levels were not altered, the redistribution of the TRAIL receptors into the lipid rafts may be of great importance for inducing apoptosis^[26]^. Using a discontinuous sucrose density gradient, we isolated lipid rafts, which were found in fractions 5-7, whereas the non-lipid rafts were found in fractions 8-10. In the parental A549 cells, TRAIL-R1 was mainly localized in the non-lipid rafts, while TRAIL-R2 was distributed in both the lipid and non-lipid rafts [Figure 5B]. The A549BTZR cells demonstrated a clear shift of TRAIL-R1 into the lipid rafts compared to the TRAIL-R1 localization in A549. Together, this finding links the lipid raft localization of TRAIL-R1 with the observed TRAIL-R1 sensitivity in the A549BTZR cells and may be mainly responsible for the reversed TRAIL sensitivity in these cells.

### TRAIL-induced apoptosis in A549BTZR cells is partially regulated by the intrinsic pathway, independent of Bcl-xL

**Figure 6:**
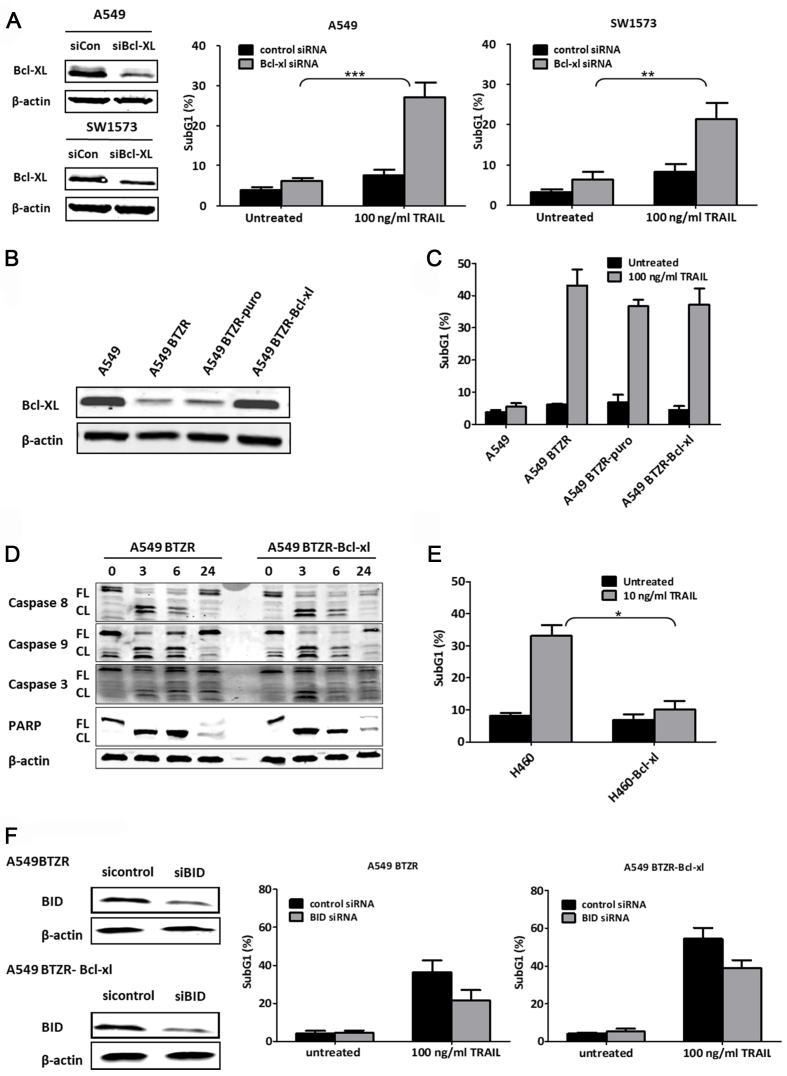
The intrinsic apoptotic pathway was involved in the TRAIL-induced apoptosis in the A549BTZR cells, though reduced levels of Bcl-xL were not a determinant. (A) The silencing of Bcl-xL, as shown by western blot analysis, resulted in enhanced cell death in the A549 and SW1573 cells after 24 h exposure to 100 ng/mL TRAIL; (B) A549BTZR cells were transfected with a control puromycin vector or a Bcl-xL-containing vector, as shown by western blot analysis; (C) Cell death was measured in the A549BTZR cells and the transfected variants after incubation with 100 ng/mL TRAIL for 24 h; (D) Western blot analysis was used to detect the cleavage of caspase-8, -9, -3, and PARP after exposure to 100 ng/mL TRAIL for several time intervals in the A549BTZR and A549BTZR-Bcl-xL cells; (E) Cell death was determined in the H460 and H460-Bcl-xL cells after exposure to 10 ng/mL TRAIL for 24 h; (F) Cell death was measured in A549BTZR and A549BTZR-Bcl-xL cells after silencing of BID. ^***^P < 0.001; ^**^P < 0.01; ^*^P < 0.05. TRAIL: TNF-related apoptosis-inducing ligand; TNF: tumor necrosis factor; Bcl-xL: B-cell lymphoma-extra large; BID: BH3 Interacting Domain Death Agonist.

Other mechanisms that could be involved in the reversed TRAIL sensitivity in A549BTZR cells are differences in the expression of Bcl-2 family member proteins. Interestingly, whereas the Mcl-1 and Bcl-2 expressions increased in the A549BTZR cells [Figure 2], the expression of Bcl-xL was markedly reduced. In order to investigate the role of Bcl-xL and TRAIL sensitivity in parental A549 and SW1573 cells, we silenced Bcl-xL protein expression [Figure 6A]. In both the TRAIL-treated A549 and SW1573 cells, a significant increase in cell death was detected, from 7% up to 27% (*P* < 0.001) and 22% (*P* < 0.01), respectively. We next examined whether the overexpression of Bcl-xL in the A549BTZR cells would regain TRAIL sensitivity by stably transfecting the A549BTZR cells with a vector encoding Bcl-xL to yield A549BTZR-Bcl-xL cells and control A549BTZR-puro cells. A western blot analysis confirmed the increased expression of Bcl-xL in the A549BTZR-Bcl-xL cells compared to the control vector-transfected cells [Figure 6B]. However, the sensitivity of the A549BTZR-Bcl-xL cells to 100 ng/mL TRAIL was not altered [Figure 6C]. Caspase cleavage was determined by western blot analysis, showing the cleavage of caspase-8, caspase-9, caspase-3, and PARP in both cell lines after 3 h of 100 ng/mL TRAIL exposure [Figure 6D]. To validate the functioning of the Bcl-xL encoding vector, we also transfected TRAIL-sensitive H460 cells. Exposure to 10 ng/mL TRAIL strongly reduced cell death in these cells, from 33% to 10% (*P* < 0.05), as expected [Figure 6E].

To further explore the requirement of the intrinsic apoptotic pathway for TRAIL-induced apoptosis in A549BTZR and A549BTZR-Bcl-xL cells, we silenced BID expression and observed the partial inhibition of cell death from 36% to 22% and 54% to 39% after exposure to 100 ng/mL TRAIL in the A549BTZR and A549BTZR-Bcl-xL cells, respectively [Figure 6F].

Taken together, these results indicate that both the extrinsic and intrinsic apoptotic pathways are involved in TRAIL-induced apoptosis in A549BTZR cells. Although the intrinsic apoptotic pathway in A549BTZR cells is apparently not regulated by Bcl-xL, Bcl-2 and Mcl-1, as well as other Bcl-2 family members not yet examined, might confer the phenotypic change of TRAIL sensitivity in A549BTZR cells.

## DISCUSSION

Intrinsic and acquired resistance are the main hurdles in the development of effective cancer therapies. Unfortunately, many cancer cell lines and tumors are intrinsically resistant to TRAIL^[18]^. In this study, we report an enhanced TRAIL sensitivity in acquired BTZ-resistant A549 cells, whereas the parental cells displayed a highly resistant TRAIL phenotype. This provided us with a very interesting model to further investigate the underlying mechanisms of TRAIL resistance.

In our study, we found a striking increase in both the OPG mRNA and protein expressions. OPG is known to be a soluble decoy receptor with a lower affinity towards TRAIL compared to TRAIL-R1 and -R2, and in contrast to these receptors, it does not transduce an apoptotic signal^[47-49]^. In ovarian cancer, OPG not only attenuated TRAIL-induced apoptosis via a TRAIL-binding manner, but also stimulated the activation of pro-survival Protein kinase B (Akt) signaling via integrin/FAK signaling and inhibited TRAIL-induced apoptosis^[50]^. Higher OPG expression and secretion were found in the TRAIL-sensitive H460 and A549BTZR cells compared to the resistant A549 and SW1573 cells. In addition, exogenously added OPG was not sensitive to TRAIL-induced apoptosis in the A549 and SW1573 cells. This might indicate an alternative function of OPG in addition to its role in suppressing TRAIL-induced apoptosis.

Furthermore, RT-PCR and western blot analyses of the A549BTZR cells demonstrated an increased expression of caspase-1 and caspase-4, which are considered inflammatory caspases^[51]^. Caspase-1 was first identified as an IL-1β-converting enzyme, also capable of processing IL-18, and is now classified as a cytokine processor caspase, along with caspases-4, -5 and -12^[52]^. Although caspase-1 was described to activate IL-1β, secretion of this cytokine was strongly reduced in the A549BTZR cells, whereas secretion of IL-6 and IL-8 was evidently increased. Enhanced levels of IL-6 were described to contribute to Mcl-1 upregulation and, subsequently, TRAIL resistance^[53]^. Controversially, increased levels of Mcl-1 did not trigger TRAIL resistance in the A549BTZR cells. Trametinib-induced FBW7-dependent Mcl-1 ubiquitination and degradation enhanced apoptosis in colorectal cancer cell lines^[54]^, while Tolksdorf et al. showed that Mcl-1 silencing may overcome the resistance of melanoma cells against TRAIL-armed oncolytic adenovirus^[55]^. However, our study showed that this effect is not very strong in NSCLC cells. Furthermore, the increased secretion of IL-6 and IL-8 was not sufficient to regulate TRAIL receptor and c-FLIP expression levels. This is in contrast to previous studies where IL-6 and IL-8 have been reported to increase c-FLIP expression levels and IL-8 was shown to reduce TRAIL-R1 expression levels, resulting in decreased TRAIL sensitivity^[56-58]^. These data indicate a complex tumor-cell-dependent interaction between TRAIL and interleukins, which may be different in NSCLC cells and needs to be investigated more in depth. Previous studies revealed that TRAIL by itself can increase levels of IL-8 via caspase-1 and -8 activation, thereby attenuating cell death^[59,60]^, or via TRAIL-R1 (which is enhanced in the presence of TRAF2 and Bcl-xL)^[61]^. In addition, BTZ increased the IL-8 level in ovarian cancer cells^[62]^ and in human monocytes and macrophages in vitro, thereby also inhibiting the expression of IL-6 and IL-1^[63]^, which may explain why its expression was increased in our study. A similar observation was made in prostate cancer cell lines in which BTZ treatment significantly increased IL-8 levels mediated by IκB kinase (IKK)α. The inhibition of this kinase enhanced the BTZ-induced p65 recruitment to the IL-8 promoter and subsequently increased IL-8 expression^[64]^. Similar findings were noted also in ovarian cancer cell lines^[65]^. The decrease in the IL-10 level in our study may also be stimulated by BTZ, as it was previously found in cutaneous T-cell lymphoma cells^[66]^. Another finding concerning BTZ is that it increased caspase-8, -9 and -3 activities in head and neck cancer cells and in gastric cancer cells^[67,68]^. Interestingly, the increase in caspase-3 may be mediated by the interaction with transmembrane and tetratricopeptide repeat-containing 2 (TMTC2) in the lipid raft domains. This molecule, enhancing the response for TRAIL, may be considered a potential predictive biomarker^[69]^.

Exploring the role of secreted cytokines on TRAIL-induced apoptosis demonstrated that the A549 parental cells did not secrete any factors to inhibit TRAIL-induced apoptosis. In addition, increased levels of OPG, IL-8, and IL-6 were not adequate to enhance TRAIL-induced apoptosis. Therefore, the inflammatory caspases might comprise an alternative role in TRAIL-induced apoptosis, since it has been described that the activation of caspase-4 via TRAIL-induced ER stress is required for enhanced caspase-3 activation^[70]^. Therefore, the contribution of ER stress in A549BTZR cells needs further exploration. Currently, more functions of caspase-1 include the activation of NF-κB^[71]^ and induction of pyroptosis, a pathway of programmed cell death distinct from apoptosis, which occurs independently of caspase-8 and -3^[72]^. However, due to the high levels of both caspase-8 and -3 cleavage, it is unlikely that A549BTZR cells are subjected to pyroptosis, while another study showed that TRAIL induced only apoptosis but not necroptosis, supporting the important role of apoptotic mechanisms in TRAIL sensitivity^[73]^. Caspase-1 is also involved in the TRAIL signaling pathway, since specific caspase-1 inhibitors abrogated TRAIL-induced apoptosis^[74-76]^. In addition, caspase-1 was previously shown to be involved in the induction of TRAIL-R2-mediated cell death in glioma cells^[59]^. Moreover, the pro-domain of caspase-1 is translocated into the nucleus to enhance Fas-mediated death signals, most likely through enhanced caspase-8 activation or reduced nuclear NF-κB activation^[77]^. Whether caspase-1 is also involved in TRAIL-R1 signaling in NSCLC cancer cells is unknown; however, enhanced (pro)caspase-1 might play a role in TRAIL-induced apoptosis in A549BTZR cells.

The trimerization of the TRAIL receptors on the cell surface, followed by the assembly of DISC and caspase-8 cleavage, is the initial step for TRAIL-induced apoptosis via the extrinsic pathway. Together with the silencing of BID, the evident cleavage of caspase-8 in A549BTZR cells further emphasizes the role of TRAIL receptors and DISC assembly. For several cell lines, including NSCLC cells and in our study, the cytotoxic effect of TRAIL receptors was most efficient when distributed in lipid rafts^[22,23,78]^, while we also demonstrated that TRAIL sensitivity was particularly induced via TRAIL-R1, which was redistributed towards the lipid rafts, similar to gastric cancer^[68]^. These findings indicate a correlation between TRAIL receptor membrane distribution and sensitivity. Interestingly, in head and neck cancer, the major player was not TRAIL-R1 but TRAIL-R2^[67]^. Based on the literature, both receptors have no significant structural differences, and in various neoplasms, the dominant mechanism of apoptosis induction may be more related to TRAIL-R1, whereas in others, instead to TRAIL-R2. There are hypotheses that the S-palmitoylation site present in TRAIL-R1 (but not in TRAIL-R2) or the GXXXG motif present in TRAIL-R2 (but not in TRAIL-R1) may be determining factors^[79,80]^. However, appropriate studies should be conducted for specific neoplasms in the future.

Upon recruitment of the DISC, pro-caspase-8 can be activated. However, some studies suggest that the dimerization and autocleavage of caspase-8 are not sufficient for full activation^[81,82]^. Active caspase-8 has been proposed to be very unstable^[82,83]^. The polyubiquitination of caspase-8 by the Cullin-3 (CUL3) E3 ligase has been shown to maintain the stability of caspase-8. Upon polyubiquitination of caspase-8, p62 is recruited to the DISC, followed by the translocation of the DISC, including the ubiquitinated caspase-8 and p62, into the cytosol to form an aggresome, which will induce cell death^[81,84]^. However, whether p62 or CUL3 is involved in the increase in TRAIL-induced apoptosis in A549BTZR cells is not yet clear. Interestingly, Huang *et al.* showed that caspase-8 cleaves BID to its truncated form, tBID^[85]^. However, in a tBID mutant knockout for the mitochondrial targeting helices (α6 and α7), apoptosis significantly decreased, indicating a crucial role of the outer mitochondrial membrane in TRAIL sensitivity. Moreover, a mutation in caspase-8 decreased TRAIL-induced apoptosis^[86]^. These data are in agreement with the predictive role of IL-8, IL-10, BID, and TNF-α in TRAIL treatment^[87,88]^. Caspase-8 may also cause TRAIL-mediated STAT1 phosphorylation and the induction of IFN-related genes^[89]^.

In addition to the trimerization of TRAIL receptors in lipid rafts, TRAIL receptors can also trimerize when distributed in non-lipid rafts. This is often correlated to the activation of the secondary complex consisting of FADD and RIP, which initiates cell survival via the activation of NF-κB. Moreover, an enhancement of the NF-κB activity upon TRAIL exposure has been described for A549 cells^[90,91]^. Therefore, one might speculate that enhanced TRAIL sensitivity in A549BTZR cells was promoted by increased levels of caspase-8 activation in combination with reduced NF-κB activity. However, NF-κB activity induced by TRAIL was also found in TRAIL-sensitive H460 cells^[32]^. In addition, Yang *et al.* described that resistance to the combination of TRAIL and BTZ in A549 spheroid models was Bcl-2-dependent and not induced by NF-κB activity^[92]^, thereby questioning the relevance of NF-κB activity in TRAIL resistance. However, in ovarian cancer, it was demonstrated that the inhibition of the NF-κB (p65) pathway via resveratrol significantly increased the efficiency of TRAIL-induced apoptosis in NSCLC, while pro-survival effects by NF-κB, Akt, and ERK(1/2) and anti-apoptosis actions by Six1 inhibited the TRAIL-R1/TRAIL-R2 pathways^[93,94]^. Moreover, NF-κB-regulated microRNAs stimulated resistance to TRAIL^[95]^. CXCL1, a target for NF-κB, stimulated immune cell infiltration via paracrine signaling, leading to TRAIL resistance in PDAC^[96]^. Based on these results, it can be concluded that NF-κB may influence cellular behavior via various signaling pathways, which should be further investigated.

Bcl-2 family members have already been identified to hamper TRAIL-induced apoptosis via the intrinsic apoptotic pathway^[38,92]^. Knockdown experiments of BID revealed that TRAIL-induced apoptosis in A549BTZR cells was also partially regulated by the intrinsic pathway and not only exerted via the extrinsic apoptotic pathway.

We further explored the role of reduced Bcl-xL expression in TRAIL sensitivity. However, although silencing of Bcl-xL sensitizes parental A549 for TRAIL-induced apoptosis, low basal levels of Bcl-xL in A549BTZR cells were not responsible for enhanced TRAIL sensitivity. The importance of Bcl-xL in TRAIL resistance appears to be variable in different cancer types (NSCLC, pancreatic cancer, glioma) and depends on the apoptotic trigger(s) applied^[93,97-100]^. In addition to Bcl-xL, other Bcl-2 family members were also differentially expressed in A549BTZR cells when compared to A549 cells. Increased levels of Bcl-2 and Mcl-1 indicate enhanced TRAIL resistance via the intrinsic apoptotic pathway. Nevertheless, NOXA might counteract enhanced Mcl-1 protein levels, e.g., by relocalization of Mcl-1 to mitochondria, an effect which needs further investigation^[101]^. In addition to NOXA, TRAIL-induced activation of p38 and JNK may also counteract the effect of Mcl-1 expression^[102]^. The mRNA levels of the pro-survival protein Bcl-2-A1 were reduced in A549BTZR cells, which may stimulate proapoptotic signaling and sensitivity to TRAIL^[103]^. Therefore, the differences identified in the expression of proteins active in the intrinsic apoptotic pathway together might tip the balance towards TRAIL-induced apoptosis.

Taken together, an abundant number of alterations were found in the A549BTZR compared to the A549 parental cells. A limitation of the study is that these aberrations were found in cells made resistant to BTZ and were not found in all resistant variants. These alterations might indicate increased levels of TRAIL resistance, such as increased Mcl-1 and Bcl-2 expression levels and enhanced secretion of OPG, IL-6, and IL-8. Nonetheless, A549BTZR cells show a TRAIL-sensitive phenotype. Therefore, the combination of several modulations contributes to TRAIL sensitivity, such as the (1) involvement of the immune system, with the enhanced activation of caspase-1 and -4; (2) activation of the extrinsic apoptotic pathway, which might be caused by TRAIL-R1 redistribution into lipid rafts; and (3) triggering of the intrinsic apoptotic pathway via alterations in the expression levels of multiple Bcl-2 family members. Further clarification regarding these mechanisms of action would provide better insight into TRAIL sensitivity and might provide novel therapeutic targets to reverse TRAIL resistance in NSCLC; (4) BID seems to be a major factor contributing to TRAIL-induced apoptosis with more influence than Bcl-2 and Mcl-1.

## DECLARATIONS

### Copyright

© The Author(s) 2024.

## References

[1] Niemira M, Collin F, Szalkowska A (2019). Molecular signature of subtypes of non-small-cell lung cancer by large-scale transcriptional profiling: identification of key modules and genes by weighted gene co-expression network analysis (WGCNA). Cancers.

[2] Testa U, Castelli G, Pelosi E (2018). Lung cancers: molecular characterization, clonal heterogeneity and evolution, and cancer stem cells. Cancers.

[3] Spira A, Ettinger DS (2004). Multidisciplinary management of lung cancer. N Engl J Med.

[4] (2018). van den Bulk J, Verdegaal EM, de Miranda NF. Cancer immunotherapy: broadening the scope of targetable tumours. Open Biol.

[5] Gotwals P, Cameron S, Cipolletta D (2017). Prospects for combining targeted and conventional cancer therapy with immunotherapy. Nat Rev Cancer.

[6] Das S, Shukla N, Singh SS, Kushwaha S, Shrivastava R (2021). Mechanism of interaction between autophagy and apoptosis in cancer. Apoptosis.

[7] Singh R, Letai A, Sarosiek K (2019). Regulation of apoptosis in health and disease: the balancing act of BCL-2 family proteins. Nat Rev Mol Cell Biol.

[8] Snajdauf M, Havlova K, Vachtenheim J Jr (2021). The TRAIL in the treatment of human cancer: an update on clinical trials. Front Mol Biosci.

[9] Singh D, Tewari M, Singh S, Narayan G (2021). Revisiting the role of TRAIL/TRAIL-R in cancer biology and therapy. Future Oncol.

[10] Carneiro BA, El-Deiry WS (2020). Targeting apoptosis in cancer therapy. Nat Rev Clin Oncol.

[11] (2019). de Looff M, de Jong S, Kruyt FAE. Multiple interactions between cancer cells and the tumor microenvironment modulate TRAIL signaling: implications for TRAIL receptor targeted therapy. Front Immunol.

[12] Herbst RS, Eckhardt SG, Kurzrock R (2010). Phase I Dose-escalation study of recombinant human Apo2L/TRAIL, a dual proapoptotic receptor agonist, in patients with advanced cancer. J Clin Oncol.

[13] Cheah CY, Belada D, Fanale MA (2015). Dulanermin with rituximab in patients with relapsed indolent B-cell lymphoma: an open-label phase 1b/2 randomised study. Lancet Haematol.

[14] Ouyang X, Shi M, Jie F (2018). Phase III study of dulanermin (recombinant human tumor necrosis factor-related apoptosis-inducing ligand/Apo2 ligand) combined with vinorelbine and cisplatin in patients with advanced non-small-cell lung cancer. Invest New Drugs.

[15] Soria JC, Márk Z, Zatloukal P (2011). Randomized phase II study of dulanermin in combination with paclitaxel, carboplatin, and bevacizumab in advanced non-small-cell lung cancer. J Clin Oncol.

[16] Oh YT, Sun SY (2021). Regulation of cancer metastasis by TRAIL/death receptor signaling. Biomolecules.

[17] Kaur I, Behl T, Sachdeva M, Bungau S, Venkatachalam T (2021). Exploring the mitochondrial apoptotic cell death landscape and associated components serving as molecular targets, primarily for synthetic and natural drugs targeting oncology therapeutics. Curr Mol Pharmacol.

[18] Stegehuis JH, de Wilt LH, de Vries EG, Groen HJ, de Jong S, Kruyt FA (2010). TRAIL receptor targeting therapies for non-small cell lung cancer: current status and perspectives. Drug Resist Updat.

[19] Kruyt FA (2008). TRAIL and cancer therapy. Cancer Lett.

[20] Borghi A, Verstrepen L, Beyaert R (2016). TRAF2 multitasking in TNF receptor-induced signaling to NF-κB, MAP kinases and cell death. Biochem Pharmacol.

[21] Greenlee JD, Lopez-Cavestany M, Ortiz-Otero N (2021). Oxaliplatin resistance in colorectal cancer enhances TRAIL sensitivity via death receptor 4 upregulation and lipid raft localization. Elife.

[22] Ouyang W, Yang C, Liu Y (2011). Redistribution of DR4 and DR5 in lipid rafts accounts for the sensitivity to TRAIL in NSCLC cells. Int J Oncol.

[23] Song JH, Tse MCL, Bellail A (2007). Lipid rafts and nonrafts mediate tumor necrosis factor related apoptosis-inducing ligand induced apoptotic and nonapoptotic signals in non small cell lung carcinoma cells. Cancer Res.

[24] Xu L, Qu X, Luo Y (2011). Epirubicin enhances TRAIL-induced apoptosis in gastric cancer cells by promoting death receptor clustering in lipid rafts. Mol Med Rep.

[25] You C, Zhang S, Sun Y (2018). β-catenin decreases acquired TRAIL resistance in non-small-cell lung cancer cells by regulating the redistribution of death receptors. Int J Oncol.

[26] Fisher MJ, Virmani AK, Wu L (2001). Nucleotide substitution in the ectodomain of trail receptor DR4 is associated with lung cancer and head and neck cancer. Clin cancer Res.

[27] Lee SH, Shin MS, Kim HS (1999). Alterations of the DR5/TRAIL receptor 2 gene in non-small cell lung cancers. Cancer Res.

[28] Wagner KW, Punnoose EA, Januario T (2007). Death-receptor O-glycosylation controls tumor-cell sensitivity to the proapoptotic ligand Apo2L/TRAIL. Nat Med.

[29] Frese S, Brunner T, Gugger M, Uduehi A, Schmid RA (2002). Enhancement of Apo2L/TRAIL (tumor necrosis factor-related apoptosis-inducing ligand)-induced apoptosis in non-small cell lung cancer cell lines by chemotherapeutic agents without correlation to the expression level of cellular protease caspase-8 inhibitory protein. J Thorac Cardiovasc Surg.

[30] Zanca C, Garofalo M, Quintavalle C (2008). PED is overexpressed and mediates TRAIL resistance in human non-small cell lung cancer. J Cell Mol Med.

[31] Mei Y, Xie C, Xie W, Tian X, Li M, Wu M (2007). Noxa/Mcl-1 balance regulates susceptibility of cells to camptothecin-induced apoptosis. Neoplasia.

[32] Voortman J, Resende TP, Abou El Hassan MA, Giaccone G, Kruyt FA (2007). TRAIL therapy in non-small cell lung cancer cells: sensitization to death receptor-mediated apoptosis by proteasome inhibitor bortezomib. Mol Cancer Ther.

[33] Manasanch EE, Orlowski RZ (2017). Proteasome inhibitors in cancer therapy. Nat Rev Clin Oncol.

[34] Orlowski RZ, Kuhn DJ (2008). Proteasome inhibitors in cancer therapy: lessons from the first decade. Clin Cancer Res.

[35] Scagliotti G (2006). Proteasome inhibitors in lung cancer. Crit Rev Oncol Hematol.

[36] (2013). de Wilt LH, Kroon J, Jansen G, de Jong S, Peters GJ, Kruyt FA. Bortezomib and TRAIL: a perfect match for apoptotic elimination of tumour cells?. Crit Rev Oncol Hematol.

[37] de Wilt LH, Jansen G, Assaraf YG (2012). Proteasome-based mechanisms of intrinsic and acquired bortezomib resistance in non-small cell lung cancer. Biochem Pharmacol.

[38] Ferreira CG, Span SW, Peters GJ, Kruyt FA, Giaccone G (2000). Chemotherapy triggers apoptosis in a caspase-8-dependent and mitochondria-controlled manner in the non-small cell lung cancer cell line NCI-H460. Cancer Res.

[39] Ashkenazi A, Pai RC, Fong S (1999). Safety and antitumor activity of recombinant soluble Apo2 ligand. J Clin Invest.

[40] Reis CR, van der Sloot AM, Natoni A (2010). Rapid and efficient cancer cell killing mediated by high-affinity death receptor homotrimerizing TRAIL variants. Cell Death Dis.

[41] van der Sloot AM, Tur V, Szegezdi E (2006). Designed tumor necrosis factor-related apoptosis-inducing ligand variants initiating apoptosis exclusively via the DR5 receptor. Proc Natl Acad Sci U S A.

[42] Cao L, Mu W (2021). Necrostatin-1 and necroptosis inhibition: pathophysiology and therapeutic implications. Pharmacol Res.

[43] Franken NAP, Rodermond HM, Stap J, Haveman J, van Bree C (2006). Clonogenic assay of cells *in vitro*. Nat Protoc.

[44] Cillessen SAGM, Meijer CJLM, Ossenkoppele GJ (2006). Human soluble TRAIL/Apo2L induces apoptosis in a subpopulation of chemotherapy refractory nodal diffuse large B-cell lymphomas, determined by a highly sensitive *in vitro* apoptosis assay. Br J Haematol.

[45] Bijnsdorp IV, Kruyt FA, Gokoel S, Fukushima M, Peters GJ (2008). Synergistic interaction between trifluorothymidine and docetaxel is sequence dependent. Cancer Sci.

[46] Zheng D, Liwinski T, Elinav E (2020). Inflammasome activation and regulation: toward a better understanding of complex mechanisms. Cell Discov.

[47] Rachner TD, Benad P, Rauner M (2009). Osteoprotegerin production by breast cancer cells is suppressed by dexamethasone and confers resistance against TRAIL-induced apoptosis. J Cell Biochem.

[48] Zinonos I, Labrinidis A, Lee M (2011). Anticancer efficacy of Apo2L/TRAIL is retained in the presence of high and biologically active concentrations of osteoprotegerin in vivo. J Bone Miner Res.

[49] Soto-Gamez A, Wang Y, Zhou X, Seras L, Quax W, Demaria M (2022). Enhanced extrinsic apoptosis of therapy-induced senescent cancer cells using a death receptor 5 (DR5) selective agonist. Cancer Lett.

[50] Lane D, Matte I, Laplante C, Garde-Granger P, Rancourt C, Piché A (2013). Osteoprotegerin (OPG) activates integrin, focal adhesion kinase (FAK), and Akt signaling in ovarian cancer cells to attenuate TRAIL-induced apoptosis. J Ovarian Res.

[51] Yazdi AS, Guarda G, D’Ombrain MC, Drexler SK (2010). Inflammatory caspases in innate immunity and inflammation. J Innate Immun.

[52] Fantuzzi G, Dinarello CA (1999). Interleukin-18 and interleukin-1 beta: two cytokine substrates for ICE (caspase-1). J Clin Immunol.

[53] Kobayashi S, Werneburg NW, Bronk SF, Kaufmann SH, Gores GJ (2005). Interleukin-6 contributes to Mcl-1 up-regulation and TRAIL resistance via an Akt-signaling pathway in cholangiocarcinoma cells. Gastroenterology.

[54] Lin L, Ding D, Xiao X, Li B, Cao P, Li S (2020). Trametinib potentiates TRAIL-induced apoptosis via FBW7-dependent Mcl-1 degradation in colorectal cancer cells. J Cell Mol Med.

[55] Tolksdorf B, Zarif S, Eberle J (2021). Silencing of Mcl-1 overcomes resistance of melanoma cells against TRAIL-armed oncolytic adenovirus by enhancement of apoptosis. J Mol Med.

[56] Abdollahi T, Robertson NM, Abdollahi A, Litwack G (2003). Identification of interleukin 8 as an inhibitor of tumor necrosis factor-related apoptosis-inducing ligand-induced apoptosis in the ovarian carcinoma cell line OVCAR3. Cancer Res.

[57] Perez LE, Parquet N, Shain K (2008). Bone marrow stroma confers resistance to Apo2 ligand/TRAIL in multiple myeloma in part by regulating c-FLIP. J Immunol.

[58] Wilson C, Wilson T, Johnston PG, Longley DB, Waugh DJ (2008). Interleukin-8 signaling attenuates TRAIL- and chemotherapy-induced apoptosis through transcriptional regulation of c-FLIP in prostate cancer cells. Mol Cancer Ther.

[59] Choi C, Kutsch O, Park J, Zhou T, Seol DW, Benveniste EN (2002). Tumor necrosis factor-related apoptosis-inducing ligand induces caspase-dependent interleukin-8 expression and apoptosis in human astroglioma cells. Mol Cell Biol.

[60] Levina V, Marrangoni AM, DeMarco R, Gorelik E, Lokshin AE (2008). Multiple effects of TRAIL in human carcinoma cells: induction of apoptosis, senescence, proliferation, and cytokine production. Exp Cell Res.

[61] Zhou DH, Yang LN, Roder C, Kalthoff H, Trauzold A (2013). TRAIL-induced expression of uPA and IL-8 strongly enhanced by overexpression of TRAF2 and Bcl-xL in pancreatic ductal adenocarcinoma cells. Hepatobiliary Pancreat Dis Int.

[62] Singha B, Phyo SA, Gatla HR, Vancurova I

[63] Sanacora S, Urdinez J, Chang TP, Vancurova I (2015). Anticancer drug bortezomib increases interleukin-8 expression in human monocytes. Biochem Biophys Res Commun.

[64] Manna S, Singha B, Phyo SA (2013). Proteasome inhibition by bortezomib increases IL-8 expression in androgen-independent prostate cancer cells: the role of IKKα. J Immunol.

[65] Singha B, Gatla HR, Manna S (2014). Proteasome inhibition increases recruitment of IκB kinase β (IKKβ), S536P-p65, and transcription factor EGR1 to interleukin-8 (IL-8) promoter, resulting in increased IL-8 production in ovarian cancer cells. J Biol Chem.

[66] Chang TP, Poltoratsky V, Vancurova I (2015). Bortezomib inhibits expression of TGF-β1, IL-10, and CXCR4, resulting in decreased survival and migration of cutaneous T cell lymphoma cells. J Immunol.

[67] Bullenkamp J, Raulf N, Ayaz B (2014). Bortezomib sensitises TRAIL-resistant HPV-positive head and neck cancer cells to TRAIL through a caspase-dependent, E6-independent mechanism. Cell Death Dis.

[68] Bui HTT, Le NH, Le QA, Kim SE, Lee S, Kang D (2019). Synergistic apoptosis of human gastric cancer cells by bortezomib and TRAIL. Int J Med Sci.

[69] Raman D, Tay P, Hirpara JL, Liu D, Pervaiz S (2021). TRAIL sensitivity of nasopharyngeal cancer cells involves redox dependent upregulation of TMTC2 and its interaction with membrane caspase-3. Redox Biol.

[70] Mao ZG, Jiang CC, Yang F, Thorne RF, Hersey P, Zhang XD (2010). TRAIL-induced apoptosis of human melanoma cells involves activation of caspase-4. Apoptosis.

[71] Lamkanfi M, Kalai M, Saelens X, Declercq W, Vandenabeele P (2004). Caspase-1 activates nuclear factor of the κ-enhancer in B cells independently of its enzymatic activity. J Biol Chem.

[72] Bergsbaken T, Fink SL, Cookson BT (2009). Pyroptosis: host cell death and inflammation. Nat Rev Microbiol.

[73] Nahacka Z, Svadlenka J, Peterka M (2018). TRAIL induces apoptosis but not necroptosis in colorectal and pancreatic cancer cells preferentially via the TRAIL-R2/DR5 receptor. Biochim Biophys Acta Mol Cell Res.

[74] Park IC, Park MJ, Woo SH (2001). Tumor necrosis factor-related apoptosis inducing ligand (TRAIL)-induced apoptosis is dependent on activation of cysteine and serine proteases. Cytokine.

[75] Ahmad M, Shi Y (2000). TRAIL-induced apoptosis of thyroid cancer cells: potential for therapeutic intervention. Oncogene.

[76] Mariani SM, Matiba B, Armandola EA, Krammer PH (1997). Interleukin 1β-converting enzyme related proteases/caspases are involved in TRAIL-induced apoptosis of myeloma and leukemia cells. J Cell Biol.

[77] Tatsuta T, Shiraishi A, Mountz JD (2000). The prodomain of caspase-1 enhances Fas-mediated apoptosis through facilitation of caspase-8 activation. J Biol Chem.

[78] Shirley S, Morizot A, Micheau O (2011). Regulating TRAIL receptor-induced cell death at the membrane: a deadly discussion. Recent Pat Anticancer Drug Discov.

[79] Neumann S, Bidon T, Branschädel M, Krippner-Heidenreich A, Scheurich P, Doszczak M (2012). The transmembrane domains of TNF-related apoptosis-inducing ligand (TRAIL) receptors 1 and 2 co-regulate apoptotic signaling capacity. PLoS One.

[80] (2014). van Roosmalen IAM, Quax WJ, Kruyt FAE. Two death-inducing human TRAIL receptors to target in cancer: similar or distinct regulation and function?. Biochem Pharmacol.

[81] Jin Z, Li Y, Pitti R (2009). Cullin3-based polyubiquitination and p62-dependent aggregation of caspase-8 mediate extrinsic apoptosis signaling. Cell.

[82] Pop C, Fitzgerald P, Green DR, Salvesen GS (2007). Role of proteolysis in caspase-8 activation and stabilization. Biochemistry.

[83] Johnson CE, Kornbluth S (2008). Caspase cleavage is not for everyone. Cell.

[84] Békés M, Salvesen GS (2009). The CULt of caspase-8 ubiquitination. Cell.

[85] Huang K, Zhang J, O’Neill KL (2016). Cleavage by caspase 8 and mitochondrial membrane association activate the BH3-only protein bid during TRAIL-induced apoptosis. J Biol Chem.

[86] Cui Z, Dabas H, Leonard BC, Shiah JV, Grandis JR, Johnson DE (2021). Caspase-8 mutations associated with head and neck cancer differentially retain functional properties related to TRAIL-induced apoptosis and cytokine induction. Cell Death Dis.

[87] Raulf N, El-Attar R, Kulms D (2014). Differential response of head and neck cancer cell lines to TRAIL or Smac mimetics is associated with the cellular levels and activity of caspase-8 and caspase-10. Br J Cancer.

[88] Polanski R, Vincent J, Polanska UM, Petreus T, Tang EK (2015). Caspase-8 activation by TRAIL monotherapy predicts responses to IAPi and TRAIL combination treatment in breast cancer cell lines. Cell Death Dis.

[89] Granqvist V, Holmgren C, Larsson C (2021). Induction of interferon-β and interferon signaling by TRAIL and Smac mimetics via caspase-8 in breast cancer cells. PLoS One.

[90] Aydin C, Sanlioglu AD, Bisgin A (2010). NF-κB targeting by way of IKK inhibition sensitizes lung cancer cells to adenovirus delivery of TRAIL. BMC Cancer.

[91] Lee KY, Park JS, Jee YK, Rosen GD (2002). Triptolide sensitizes lung cancer cells to TNF-related apoptosis-inducing ligand (TRAIL)-induced apoptosis by inhibition of NF-kappaB activation. Exp Mol Med.

[92] Yang TM, Barbone D, Fennell DA, Broaddus VC (2009). Bcl-2 family proteins contribute to apoptotic resistance in lung cancer multicellular spheroids. Am J Respir Cell Mol Biol.

[93] Rasheduzzaman M, Jeong JK, Park SY (2018). Resveratrol sensitizes lung cancer cell to TRAIL by p53 independent and suppression of Akt/NF-κB signaling. Life Sci.

[94] Yang J, Li G, Zhang K (2016). Pro-survival effects by NF-κB, Akt and ERK(1/2) and anti-apoptosis actions by Six1 disrupt apoptotic functions of TRAIL-Dr4/5 pathway in ovarian cancer. Biomed Pharmacother.

[95] Jeon YJ, Middleton J, Kim T (2021). Correction for Jeon et al., a set of NF-κB-regulated microRNAs induces acquired TRAIL resistance in lung cancer. Proc Natl Acad Sci U S A.

[96] Geismann C, Erhart W, Grohmann F (2018). TRAIL/NF-κB/CX3CL1 mediated onco-immuno crosstalk leading to TRAIL resistance of pancreatic cancer cell lines. Int J Mol Sci.

[97] Cingöz A, Ozyerli-Goknar E, Morova T (2021). Generation of TRAIL-resistant cell line models reveals distinct adaptive mechanisms for acquired resistance and re-sensitization. Oncogene.

[98] Kim J, Yun M, Kim EO (2016). Decursin enhances TRAIL-induced apoptosis through oxidative stress mediated- endoplasmic reticulum stress signalling in non-small cell lung cancers. Br J Pharmacol.

[99] Chen M, Wang X, Zha D (2016). Apigenin potentiates TRAIL therapy of non-small cell lung cancer via upregulating DR4/DR5 expression in a p53-dependent manner. Sci Rep.

[100] Hinz S, Trauzold A, Boenicke L (2000). Bcl-XL protects pancreatic adenocarcinoma cells against CD95- and TRAIL-receptor-mediated apoptosis. Oncogene.

[101] Nakajima W, Hicks MA, Tanaka N, Krystal GW, Harada H (2014). Noxa determines localization and stability of MCL-1 and consequently ABT-737 sensitivity in small cell lung cancer. Cell Death Dis.

[102] Azijli K, Yuvaraj S, van Roosmalen I (2013). MAPK p38 and JNK have opposing activities on TRAIL-induced apoptosis activation in NSCLC H460 cells that involves RIP1 and caspase-8 and is mediated by Mcl-1. Apoptosis.

[103] Unterkircher T, Cristofanon S, Vellanki SH (2011). Bortezomib primes glioblastoma, including glioblastoma stem cells, for TRAIL by increasing tBid stability and mitochondrial apoptosis. Clin Cancer Res.

