# Supplementary material for: Mechanisms underlying reversed TRAIL sensitivity in acquired bortezomib-resistant non-small cell lung cancer cells
Source: Cancer Drug Resist. 2024 Apr 9;7:12. doi: 10.20517/cdr.2024.14 (PMC11149110; doi:10.20517/cdr.2024.14)
Supplement: Supplementary file 1 [file cdr-7-12-SupplementaryMaterials.pdf]

De Wilt *et al. Cancer Drug Resist* 2024;7:12
DOI: 10.20517/cdr.2024.14

Cancer Drug Resistance

Supplementary Materials

Mechanisms underlying reversed TRAIL sensitivity in acquired bortezomib-resistant non-small cell lung cancer cells

Leonie De Wilt[1,#], Bartosz Kamil Sobocki[2,#], Gerrit Jansen[3], Hessan Tabeian[1], Steven de Jong[4], Godefridus J. Peters[1,2], Frank Kruyt[4]

[1]Department of Medical Oncology, Amsterdam University Medical Centers, Location VUMC, Vrije Universiteit Amsterdam, Amsterdam 1007MB, the Netherlands.

[2]Department of Biochemistry, Medical University of Gdańsk, Gdańsk 80-210, Poland.

[3]Department of Rheumatology, Amsterdam University Medical Centers, Vrije Universiteit Amsterdam, Amsterdam 1081 HV, the Netherlands.

[4]Department of Medical Oncology, University of Groningen, University Medical Center Groningen, Groningen 9713 GZ, the Netherlands.

[#]Authors contributed equally.

Correspondence to: Prof. Godefridus J. Peters, Department of Biochemistry, Medical University of Gdańsk, M. Skłodowskiej-Curie 3a street, Gdańsk 80-210, Poland. E-mail: gj.peters@gumed.edu.pl; Prof. Frank Kruyt, Department of Medical Oncology, University of Groningen, University Medical Center Groningen, Hanzeplein 1, Groningen 9713 GZ, the Netherlands. E-mail: f.a.e.kruyt@umcg.nl

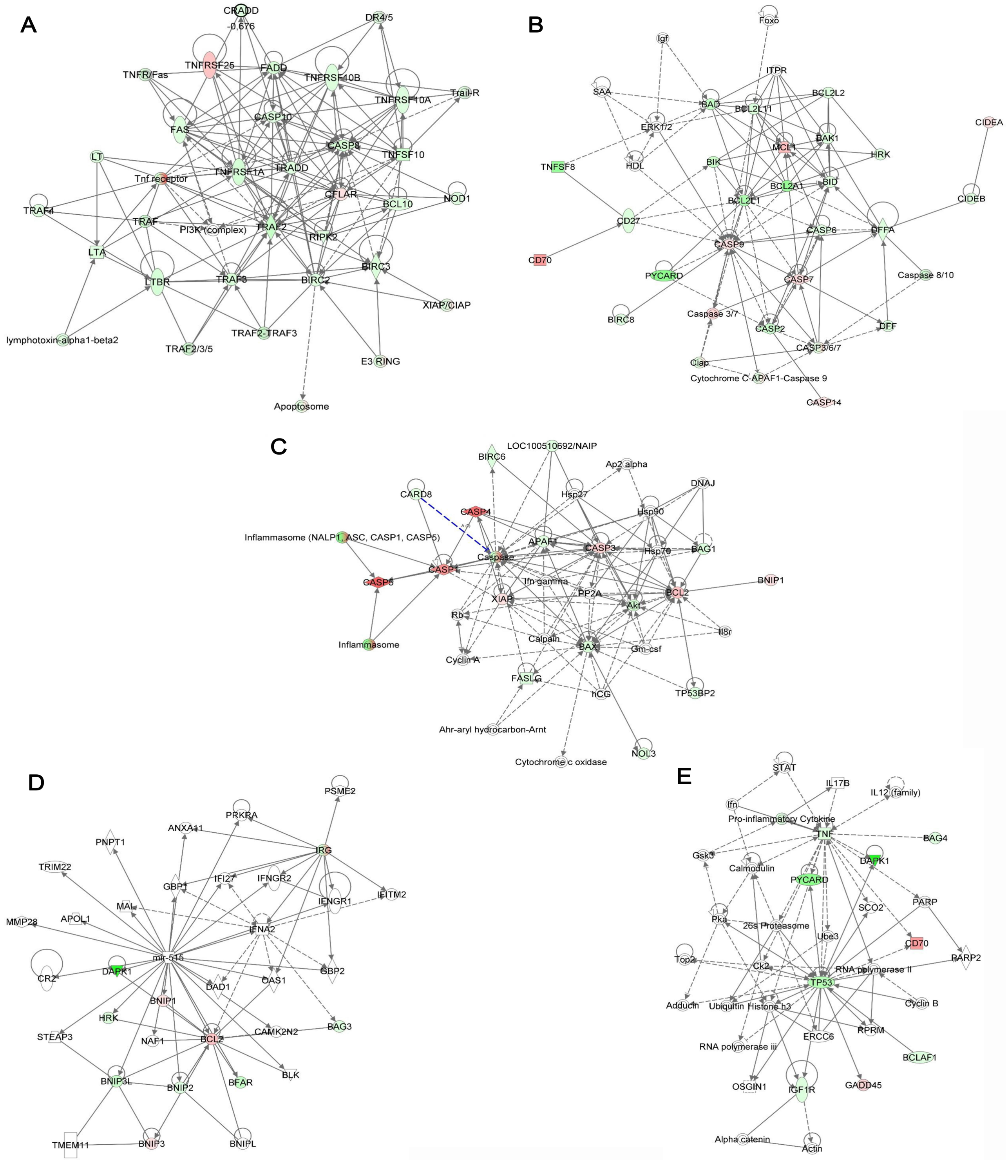

Supplementary Figure 1. Green is downregulated in A549BTZR, Red is upregulated in A549BTZR, White is unknown The stronger the color the more the target is up or down regulated.
